# Validation of a novel system to assess end-expiratory lung volume and alveolar recruitment in an ARDS model

**DOI:** 10.1186/s40635-021-00410-x

**Published:** 2021-09-10

**Authors:** Laurent Bitker, Nadja Cristinne Carvalho, Sascha Reidt, Christoph Schranz, Dominik Novotni, Maciej Orkisz, Eduardo Davila Serrano, Jean-Pierre Revelly, Jean-Christophe Richard

**Affiliations:** 1 Service de Médecine Intensive - Réanimation, Hôpital De La Croix Rousse, Hospices Civils de Lyon, 103 Grande Rue de la Croix Rousse, 69004 Lyon, France; 2 Université de Lyon, Université Claude Bernard Lyon 1, INSA-Lyon, UJM-Saint Etienne, CNRS, Inserm, CREATIS UMR 5220, U1206, Villeurbanne, France; 3 Research and New Technology Department, Hamilton Medical AG, Bonaduz, Switzerland

**Keywords:** Acute respiratory distress syndrome, Positive end-expiratory pressure, Lung injury, End-expiratory lung volume, Functional residual capacity, Computed tomography, Alveolar recruitment, Nitrogen washin–washout

## Abstract

**Background:**

Personalizing mechanical ventilation requires the development of reliable bedside monitoring techniques. The multiple-breaths nitrogen washin–washout (MBNW) technique is currently available to measure end-expiratory lung volume (EELV_MBNW_), but the precision of the technique may be poor, with percentage errors ranging from 28 to 57%. The primary aim of the study was to evaluate the reliability of a novel MBNW bedside system using fast mainstream sensors to assess EELV in an experimental acute respiratory distress syndrome (ARDS) model, using computed tomography (CT) as the gold standard. The secondary aims of the study were: (1) to evaluate trending ability of the novel system to assess EELV; (2) to evaluate the reliability of estimated alveolar recruitment induced by positive end-expiratory pressure (PEEP) changes computed from EELV_MBNW_, using CT as the gold standard.

**Results:**

Seven pigs were studied in 6 experimental conditions: at baseline, after experimental ARDS and during a decremental PEEP trial at PEEP 16, 12, 6 and 2 cmH_2_O. EELV was computed at each PEEP step by both the MBNW technique (EELV_MBNW_) and CT (EELV_CT_). Repeatability was assessed by performing replicate measurements. Alveolar recruitment between two consecutive PEEP levels after lung injury was measured with CT (Vrec_CT_), and computed from EELV measurements (Vrec_MBNW_) as ΔEELV minus the product of ΔPEEP by static compliance.

EELV_MBNW_ and EELV_CT_ were significantly correlated (*R*^2^ = 0.97). An acceptable non-constant bias between methods was identified, slightly decreasing toward more negative values as EELV increased. The conversion equation between EELV_MBNW_ and EELV_CT_ was: EELV_MBNW_ = 0.92 × EELV_CT_ + 36. The 95% prediction interval of the bias amounted to ± 86 mL and the percentage error between both methods amounted to 13.7%.

The median least significant change between repeated measurements amounted to 8% [CI_95%_: 4–10%]. EELV_MBNW_ adequately tracked EELV_CT_ changes over time (concordance rate amounting to 100% [CI_95%_: 87%–100%] and angular bias amounting to − 2° ± 10°).

Vrec_MBNW_ and Vrec_CT_ were significantly correlated (*R*^2^ = 0.92). A non-constant bias between methods was identified, slightly increasing toward more positive values as Vrec increased.

**Conclusions:**

We report a new bedside MBNW technique that reliably assesses EELV in an experimental ARDS model with high precision and excellent trending ability.

**Supplementary Information:**

The online version contains supplementary material available at 10.1186/s40635-021-00410-x.

## Background

Despite improvement in therapeutic management, acute respiratory distress syndrome (ARDS) mortality remains high in observational studies [1]. Personalizing mechanical ventilation may improve ARDS prognosis [2], but requires the development of reliable bedside monitoring techniques. End-expiratory lung volume (EELV) monitoring is appealing as it provides information on the size of the baby lung and lung strain, and may help individualize respiratory settings during mechanical ventilation of ARDS patients, by estimating alveolar recruitment related to positive end-expiratory pressure (PEEP) changes [3]. However, this clinical application has not been validated so far, using computed tomography (CT) as the reference technique.

The multiple-breaths nitrogen washin–washout (MBNW) technique is currently available on some ICU ventilators, and has been shown reliable to assess EELV in both mechanically ventilated patients at PEEP 5 cmH_2_O [4] and in experimental studies at higher PEEP [5, 6], using CT as the reference technique to measure EELV. However, the precision of the technique may be poor, with percentage errors ranging from 28 to 57%, and is accompanied with a systematic bias at high PEEP levels identified in one study [6]. Such bias at high PEEP levels was merit of attention of a previous study, where the presence of overinflated lung region and increased alveolar dead space at higher PEEP levels partially explained the substantial difference between EELV measured by CT and MBNW [7]. A new non-invasive MBNW monitor was designed, using fast mainstream sensors enabling continuous and synchronous measurements of proximal flow and O_2_/CO_2_ concentrations. We hypothesized that this new system might be more reliable to assess EELV.

The aim of the study was primarily to evaluate the reliability of this new non-invasive MBNW monitor to measure EELV at bedside (bias, precision, percentage error, and repeatability) in an experimental model of ARDS using CT as the gold standard. Secondly, the trending ability of the new MBNW monitor and the reliability of estimated alveolar recruitment induced by PEEP changes computed from EELV_MBNW_ measurements were evaluated, using CT as the gold standard.

## Methods

The study was approved by an Institutional Review Board for the care of animal subjects (Comité d’éthique en experimentation animale n°042, APAFIS#21542–2019071909353561), and carried out on 7 female Large White pigs weighing 29 [29, 30] kg, in accordance with Good Laboratory Practice Regulation [8].

### Animal preparation

Pigs were premedicated with an intramuscular injection of xylazine 2% (0.7 mg kg^−1^), ketamine (17 mg kg^−1^), and droperidol (0.17 mg kg^−1^). Surgical preparation was performed in the supine position under continuous general anesthesia by propofol (7 mg kg h^−1^) and fentanyl (10 µg kg h^−1^). Body temperature was maintained constant at 38 ± 1 °C using a heating pad.

A 6.0 tracheal tube was introduced via median surgical tracheotomy, secured hermetically after cuff inflation, and connected to a HAMILTON-C6 ventilator (Hamilton Medical AG, Bonaduz, SWITZERLAND). Mechanical ventilation was initially performed through a heated humidifier (HAMILTON-H900, Hamilton Medical AG, Bonaduz, SWITZERLAND) in volume-controlled mode, constant inspiratory flow, tidal volume (V_T_) 10 mL kg^−1^, inspired fraction of oxygen (FiO_2_) 50%, PEEP 5 cm H_2_O and respiratory rate adjusted to achieve normocapnia. Muscle relaxation was obtained with cisatracurium (30 mg h^−1^).

The left jugular vein was surgically cannulated with a 3 lm 8.5 Fr catheter (model Arrow # CV-12853, Teleflex, Wayne, PA, USA) for drug administration. A 7-french pulmonary artery catheter (model #131F7, Edwards, Irvine, CA, USA) was advanced through the right external jugular vein to assess central venous pressure, pulmonary artery pressure and cardiac output. The left carotid artery was surgically cannulated with an 8.5 Fr catheter for continuous monitoring of arterial blood pressure.

### Experimental protocol (Fig. 1)

**Fig. 1:**
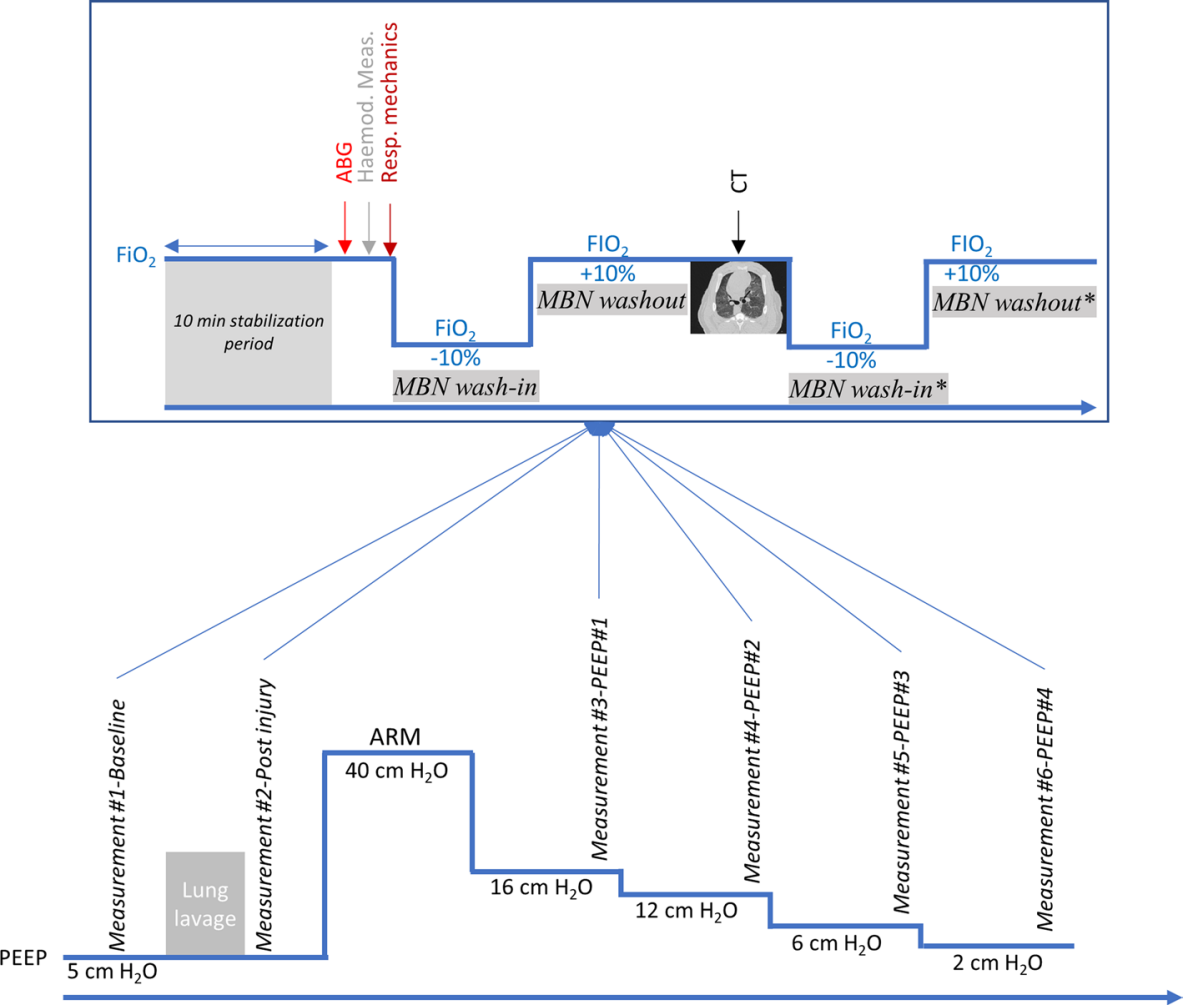
Experimental protocol. *ABG* arterial blood gas, *ARM*  alveolar recruitment maneuver, *CT* computed tomography, *FiO*_*2*_ fraction of inspired oxygen, *Hemod*. *Meas*. hemodynamic measurements, *MBN* multiple breath nitrogen, *PEEP* positive end-expiratory pressure; *Resp.* *mechanics* respiratory mechanics measurements. * refers to MBNW replicates performed in all experimental conditions on 5 pigs of the study

Experimental ARDS was achieved by saline lavage under FiO_2_ 90%, VT 10 ml.kg^−1^ and PEEP 5 cm H_2_O. Repeated intra-tracheal instillations of 1000 mL 0.9% sodium chloride warmed at 37 °C was performed, and lung fluid was removed by both gravity and endotracheal suctioning. The procedure aimed to achieve a PaO_2_/FiO_2_ ratio < 100 mm Hg 15 min after the last lung lavage. Respiratory rate (RR) could be increased up to 30 breaths per min to maintain pH above 7.20. A recruitment maneuver was then executed by applying a continuous airway pressure of 40 cm H_2_O during 20 s, VT was set to 6 ml.kg^−1^, FiO_2_ was set to 80%, and a decremental PEEP trial (16, 12, 6, and 2 cm H_2_O) was performed. PEEP could possibly be increased by 1 cm H_2_O increments at the last two PEEP steps if the peripheral oxygen saturation fell below 88% to maintain the animal alive.

The following measurements were successively performed at each protocol step, i.e., at baseline, 15 min after the achievement of experimental ARDS criteria, and after a 10-min stabilization period at each PEEP step (Fig. 1): arterial blood gas, hemodynamic measurements, respiratory mechanics assessment, first assessment of EELV by MBNW, assessment of EELV by CT, and ultimately replicate assessment of EELV by MBNW.

### Measurements

#### Arterial blood gas

Arterial blood gases were analyzed with an ABL80 FLEX CO-OX blood gas analyzer (Radiometer, Copenhagen, DENMARK) for oxygen and carbon dioxide partial pressures, pH and lactate.

#### Hemodynamic measurements

Pressure transducers (Abbott, San Clemente, CA, USA) were positioned at the mid-chest level and connected to a Philips monitor (M1205A Philips Healthcare, Best, NETHERLAND). Cardiac output was measured in triplicate with the thermodilution technique, using 10 ml saline injected at room temperature.

#### Respiratory measurements

Airway pressure and flow were measured by the HAMILTON proximal flow sensor of the mechanical ventilator. Total PEEP and plateau pressure were measured at the end of 3 s end-expiratory and end-inspiratory pauses, respectively. Peripheral capillary oxygen saturation was measured continuously on the tip of the animal tong.

#### *Multiple-breaths nitrogen washin–washout determination of end-expiratory lung volume (EELV*_*MBNW*_*)*

EELV_MBNW_ was determined using a MBNW maneuver, consisting of a change in FiO_2_ by 10% and reaching a new steady-state, before resetting the FiO_2_ to the original value. A novel mainstream CO_2_/O_2_ gas sensor was placed between the proximal flow sensor and the tracheal tube, measuring O_2_/CO_2_ partial pressures at a frequency of 100 Hz. Gas flow, airway pressure and O_2_/CO_2_ partial pressures were acquired by the ventilator and relayed via a serial connection to a computer running a custom MATLAB program (MathWorks, Natick, MA, USA). The signals were processed online and automatically calculating the breath-by-breath volumetric gas exchange [9]. The cumulative volume of nitrogen washed-out by the increase in FiO_2_ was determined by integrating gas flow, O_2_ and CO_2_ partial pressure signals and subtracting the nitrogen baseline determined during the steady-states. The cumulative volume of nitrogen retained during the wash-in phase was computed accordingly. EELV_MBNW_ was then computed as:

$${\mathrm{EELV}}_{\mathrm{MBNW}}= \frac{\mathrm{Cumulative} \Delta \mathrm {N}2\mathrm{ volume }}{\Delta \mathrm{N}2\mathrm{ fraction}}.$$

EELV_MBNW_ measured during wash-in and wash-out were averaged. To allow meaningful comparisons with EELV_CT_, instrumental dead-space, i.e., the volume from the O_2_/CO_2_ sensor to the tip of the tracheal tube was measured and subtracted from EELV_MBNW_. The stability of carbon dioxide production (VCO_2_) and oxygen consumption (VO_2_) before and after the MBNW maneuver was verified. Repeated EELV_MBNW_ measurements (i.e., two consecutives nitrogen washin–washout) at each PEEP level were carried out in five pigs of the study to assess repeatability of the technique.

An estimation of the recruited volume between two consecutive PEEP levels after lung injury was performed with the MBNW technique (Vrec_MBNW_) as follows [3]:

$${\mathrm{Vrec}}_{\mathrm{MBNW}}={(\mathrm{EELV}}_{\mathrm{PEEPn}+1}- {\mathrm{EELV}}_{\mathrm{PEEPn}})-{\mathrm{Compliance}}_{\mathrm{LowerPEEP}}\bullet ({\mathrm{PEEP}}_{n+1}-{\mathrm{PEEP}}_{n}),$$

with *n* = rank of measurement and EELV assessed with MBNW technique.

#### CT measurements

EELV_CT_ was measured on a Biograph mCT (Siemens, Munich, GERMANY) previously calibrated with the manufacturer phantom, using the following settings: voltage 120 kV, pitch 1.2, current time-product 80 mAs, field-of-view diameter 500 mm, slice thickness 1 mm, Kernel B31f (smooth), matrix size 512 by 512, and pixel size 0.8 mm by 0.8 mm.

Lung scanning was performed from apex to base during both end-expiratory and end-inspiratory pauses, with clamped tracheal tubes. The lungs were semi-automatically segmented, followed by visual inspection by one author (JCR) with a previously validated in-house software [10], excluding pleural effusions, hilar and mediastinal structures. Segmented lung volumes were analyzed using MATLAB (MathWorks, Natick, MA, USA).

Gas and tissue volume in each lung voxel and end-expiratory lung volume (EELV_CT_) were computed from the CT number (HU) according to the following formulae [11]:

Voxel gas volume = $\frac{\mathrm{CT number}}{-1000}\bullet \mathrm{voxel volume}$ for lung voxels with − 1000 ≤ CT number ≤ 0.

Voxel gas volume = 0 for lung voxels with CT number > 0.

Voxel gas volume = voxel volume for lung voxels with CT number < – 1000.

Voxel tissue volume = voxel volume—voxel gas volume.

EELV_CT_ = $$\sum_{i=1}^{k}voxel gas volume(i)$$ with k = total number of voxels in segmented lung volume at end-expiration.

Lung parenchyma was then classified into four compartments, according to CT number [11]: non-inflated (density between + 100 and − 100 Hounsfield units (HU)), poorly inflated (density between − 101 and − 500 HU), normally inflated (density between – 501 and – 900 HU), and overinflated tissue (density between − 901 and − 1000 HU). The volume of each compartment was measured at end-expiration and end-inspiration.

CT-derived recruited volume (Vrec_CT_ in mL) between two consecutive PEEP levels after lung injury was estimated on end-expiratory CT scans as follows [12]:

$${\mathrm{Vrec}}_{\mathrm{CT}}=\frac{{\mathrm{Vtis}}_{\mathrm{ PEEPn}}-{\mathrm{Vtis}}_{\mathrm{ PEEP}n+1}}{1-{F\mathrm{gas}}_{\mathrm{PEEP}n}}\bullet {Fgas}_{\mathrm{PEEP}n},$$

with *n* = rank of measurement, ${Vtis}_{PEEPn}$= tissue volume of the non-aerated compartment at PEEP_n_ at end-expiration, ${Vtis}_{PEEPn+1}=$ tissue volume of the non-aerated compartment at PEEP_*n*+1_ at end-expiration, ${Fgas}_{PEEPn}=$ gas fraction of the aerated compartment at PEEP_n_ at end-expiration.

### Statistical analysis

Statistical analyses were performed using R for Windows v4.0.2 [13] with the following packages: boot [14, 15], lme4 [16], lmerTest [17], MuMIn [18], MethComp [19], and multcomp [20]. A p-value below 0.05 was chosen for statistical significance. Values were expressed as median [1st quartile–3rd quartile] unless otherwise stated. 95% confidence intervals (CI_95%_) of proportions were computed with the Wilson score method.

Ventilatory, respiratory, hemodynamic and CT variables were compared across experimental conditions with a linear mixed model, using experimental condition as a factor with fixed effect, and pigs as a factor with random effect to consider multiple measurements. Multiple comparisons were performed with Dunnett’s test using baseline measurements as a reference.

Repeatability of the MBNW technique was assessed as follows. The coefficient of variation (CV) of EELV_MBNW_ repeated measurements was computed as the standard deviation divided by the mean of the 2 replicates. The precision of replicates for EELV_MBNW_ measurement was computed as 2 × CV. The least significant change (LSC) between repeated measurements was computed as CV × 1.96 × $\sqrt{2} [21]$. The bias corrected and accelerated bootstrap method with 10,000 replicates was used to compute CI_95%_ for precision and LSC [22].

EELV_MBNW_ and EELV_CT_ were compared using a linear mixed-effect model, and Bland and Altman representation, using the first EELV_MBNW_ replicate [23]. Bias between methods was computed as EELV_MBNW_ minus EELV_CT_. Since bias was non-constant and the experimental design involved repeated measurements, limits of agreement and conversion equation between EELV_MBNW_ and EELV_CT_ were computed from the posterior medians of a Monte Carlo chain simulation [24]. Percentage error was computed as 2 × SD_Bias_/mean EELV_CT_ [25], with SD_Bias_ = standard deviation of the bias. Similar analyses were performed with Vrec_MBNW_ and Vrec_CT_.

To explore the impact of potential confounding variables on the bias between methods, a linear mixed-effects model was built using ventilatory, respiratory, hemodynamic and CT variables as fixed effects, pigs as factor with random effect [26], and bias between methods as the dependent variable. Variables with *p* values < 0.1 in univariate analyses were considered for inclusion in a multivariable model. Model simplification was performed using a backward stepwise algorithm.

Ability of the MBNW technique to track directional changes in EELV between experimental conditions was assessed using four-quadrant and polar plots. Concordance rate was defined as the percentage of data points falling into one of the two quadrants of agreement on the four-quadrants plot (i.e., quadrants in which both EELV_MBNW_ and EELV_CT_ have the same directional changes) [27]. Since the four-quadrant plot does not quantify the distance between each data point and the line of identity, a polar plot analysis was performed [27]. Angular bias (difference in calibration between the reference and test method) was computed as the mean angle between all data points and polar axis [27], and compared to zero using Mann–Whitney U test. Radial limits of agreement (defined as the radial sector containing 95% of the data points, after conversion of negative deflections to positive ones) were computed similarly to the limits of agreement in Bland and Altman analysis [27].

## Results

Ten measurements were missing due to various reasons, including premature death of one animal as reported in Additional file 1 and ending up in 32 available measurements. Variables repeatedly studied over time are reported in Table 1. After experimental lung injury, PaO_2_/FiO_2_, pH and EELV decreased significantly while PaCO_2_, plateau pressure and mean pulmonary artery pressure increased significantly. EELV_CT_ values spanned from 254 to 1200 mL, while EELV_MBNW_ ranged from 298 to 1157 mL.

**Table 1:** Variables repeatedly assessed over time

Variables	Baseline	Post-injury	PEEP#1	PEEP#2	PEEP#3	PEEP#4	*p* value
PEEP (cm H_2_O)	5 [5–5]	5 [5–5]	16 [16–16]^†^	12 [12–12]^†^	6 [6–8]^†^	3 [2–5]^†^	< 0.001
VT (ml kg^−1^)	10.0 [10.0–10.0]	10.0 [10.0–10.0]	5.9 [5.9–6.1]^†^	5.9 [5.9–6.1]^†^	5.9 [5.9–6.1]^†^	5.9 [5.9–6.0]^†^	< 0.001
PEEP_tot_ (cm H_2_O)	5 [5–5]	6 [5–6]	17 [16–17]^†^	13 [12–13]^†^	7 [6–8]^†^	4 [3–6]	< 0.001
PEEP_int_ (cm H_2_O)	0 [0–0]	0 [0–1]	1 [0–1]^†^	1 [0–1]^†^	0 [0–1]	1 [1–1]^†^	< 0.01
P_Plat_ (cm H_2_O)	16 [16–18]	29 [26–30]^†^	28 [26–29]^†^	22 [22–23]^†^	22 [19–22]^†^	24 [21–25]^†^	< 0.001
RR (min^−1^)	15 [15–16]	19 [15–24]	25 [25–28]^†^	25 [25–28]^†^	28 [25–28]^†^	28 [27–29]^†^	< 0.001
HR (min^−1^)	97 [86–118]	113 [108–123]	105 [100–107]	102 [97–133]	128 [109–130]	128 [117–142]	0.11
MAP (mm Hg)	84 [80–92]	92 [87–97]	95 [89–95]	86 [85–102]	104 [101–109]^†^	106 [104–108]^†^	< 0.05
MPAP (mm Hg)	24 [23–27]	32 [31–37]^†^	35 [33–39]^†^	34 [31–38]^†^	40 [37–47]^†^	41 [34–46]^†^	< 0.01
PAOP (mm Hg)	11 [11–11]	13 [12–14]^†^	14 [14–14]^†^	13 [13–13]^†^	11 [11–12]	10 [10–10]	< 0.001
CVP (mm Hg)	10 [10–12]	10 [9–14]	13 [12–13]^†^	12 [11–12]	10 [10–10]	9 [8–9]	< 0.001
CO (L.min^−1^)	3.5 [3.4–4.2]	4.4 [3.8–4.5]	2.9 [2.5–3.1]^†^	3.2 [2.8–3.3]	4.1 [3.6–4.4]	4.6 [4.3–4.8]	< 0.001
pH	7.45 [7.40–7.48]	7.28 [7.25–7.38]^†^	7.32 [7.29–7.34]^†^	7.34 [7.32–7.36]^†^	7.33 [7.28–7.36]^†^	7.25 [7.25–7.31]^†^	< 0.001
PaO_2_/FiO_2_ (mm Hg)	435 [378–466]	92 [80–96]^†^	341 [335–342]	370 [342–398]	131 [85–237]^†^	83 [66–96]^†^	< 0.001
PaCO_2_ (mm Hg)	42 [37–47]	59 [44–61]^†^	53 [51–56]^†^	52 [52–53]^†^	60 [53–61]^†^	68 [58–74]^†^	< 0.001
Lactate (mmol/L^−1^)	1.2 [1.0–1.4]	0.9 [0.8–1.1]	0.7 [0.6–1.0]	0.7 [0.5–0.9]	0.6 [0.5–0.7]	0.6 [0.5–0.8]	0.15
EELW_MBNW_ (mL)	610 [579–656]	392 [357–455]^†^	987 [901–1059]^†^	850 [831–872]^†^	466 [427–553]	363 [348–428]^†^	< 0.001
EELW_CT_ (mL)	604 [533–615]	353 [324–407]^†^	1019 [988–1122]^†^	846 [824–888]^†^	470 [463–472]^†^	323 [295–420]^†^	< 0.001

*CO* = cardiac output; *CVP* central venous pressure; *EELV*_*CT*_ end-expiratory lung volume assessed with computed tomography; *EELV*_*MBNW*_ end-expiratory lung volume assessed with the multiple breaths N_2_ washin–washout technique; *HR* heart rate; *MAP* mean arterial pressure; *MPAP* mean pulmonary artery pressure; *PaCO*_*2*_ arterial carbon dioxide partial pressure; *PaO*_*2*_ arterial oxygen partial pressure; *PAOP* pulmonary arterial occlusion pressure; *PEEP* positive end-expiratory pressure; *PEEP*_*int*_ intrinsic PEEP (i.e., PEEP_tot_-PEEP); *PEEP*_*tot*_ total PEEP; *P*_*Plat*_ plateau pressure; *RR* respiratory rate; *VT* tidal volume

Values are median [1st quartile–3rd quartile]. ^†^*p* < 0.05 vs baseline

### Repeatability of the MBNW technique

27 replicate EELV_MBNW_ measurements were obtained. The median precision of repeated EELV_MBNW_ measurements was 6% [CI_95%_: 3–7%]. The median LSC between repeated EELV_MBNW_ measurements was 8% [CI_95%_: 4–10%].

### ***Comparison of EELV***_***MBNW***_*** and EELV***_***CT***_

**Fig. 2:**
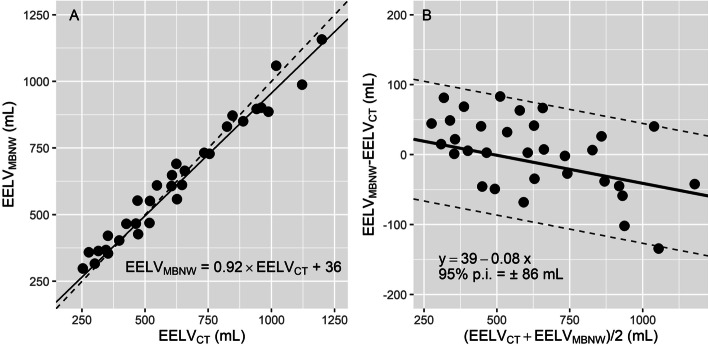
Correlation between EELV_MBNW_ and EELV_CT_ (**A**) and corresponding Bland and Altman plot (**B**). Each symbol represents a concomitant measurement of end-expiratory lung volume assessed with the multi-breath nitrogen washin–washout technique (EELV_MBNW_) and computed tomography (EELV_CT_). **A** Solid line is regression line. Dashed line is line of identity. **B** continuous line and the 2 broken lines are the mean bias and 95% prediction interval limits of the bias between methods_,_ respectively. 95% p.i. = 95% prediction interval of the bias between methods

EELV_MBNW_ and EELV_CT_ were significantly correlated (marginal *R*^2^ = 0.97, p < 0.001, Fig. 2A), and the regression equation between EELV_MBNW_ and EELV_CT_, had an intercept of 36 mL (*p* < 0.001) and a slope of 0.92. A non-constant bias between methods was identified on Bland and Altman plot (Fig. 2B), with a slight albeit significant trend of more negative values as EELV increased with PEEP. The conversion equation between EELV_MBNW_ and EELV_CT_ was computed as EELV_MBNW_ = 0.92 × EELV_CT_ + 36. The 95% prediction interval of the bias between methods amounted to ± 86 mL, and the percentage error between both methods was 13.7%.

Multivariate analysis identified the volume of the overinflated compartment at end-expiration as the only variable independently associated with the bias between methods (Table 2), with a linear coefficient amounting to – 8.7 ± 1.8 (i.e., for each mL of overinflated volume increase, the EELV_MBNW_ is 9 ml lower than the reference EELV value measured with CT).

**Table 2:** Uni- and multivariate analysis of variables associated with the bias between methods

Variables	Univariate coefficient	Univariate *p* value	Multivariate coefficient	Multivariate *p* value
PEEP (per 1 cm H_2_O increase)	− 7.2 ± 1.5	< 0.0001	–	–
VT (per 10 ml increase)	3.1 ± 1.4	< 0.05	–	–
PEEP_TOT_ (per 1 cm H_2_O increase)	− 7.0 ± 1.4	< 0.0001	–	–
P_PLAT_ (per 1 cm H_2_O increase)	− 2.1 ± 1.9	< 0.05	–	–
Δ*P* (per 1 cm H_2_O increase)	4.5 ± 1.7	< 0.01	–	–
RR (per 1 cycle.min^−1^ increase)	− 3.5 ± 1.5	< 0.05	–	–
CO (per 1 L.min^−1^ increase)	36.7 ± 9.8	< 0.05	–	–
Overinf. cpt at expiration (per 1 mL increase)	− 8.5 ± 1.7	< 0.0001	− 8.7 ± 1.8	< 0.0001
Overinf. cpt at inspiration (per 1 mL increase)	− 3.8 ± 1.1	< 0.01	–	–
Non-inf. cpt at expiration (per 1 mL increase)	0.12 ± 0.05	< 0.05	–	–

Bias between methods was computed as EELV_MBNW_ minus EELV_CT_. PEEP and Δ*P* were not included in the multivariate model for collinearity with PEEP_TOT_

*CO* cardiac output; *∆P* driving pressure *EELV*_*CT*_ end-expiratory lung volume assessed with computed tomography; *EELV*_*MBNW*_ end-expiratory lung volume assessed with the multiple breaths N_2_ washin–washout technique; *non-inf. cpt* non-inflated compartment on CT; *overinf. cpt.* overinflated compartment on CT; *PEEP* positive end-expiratory pressure; *PEEP*_*TOT*_ total positive end-expiratory pressure; *P*_*PLAT*_ plateau pressure; *RR* respiratory rate; *VT* tidal volume

Ability of the MBNW technique to track directional changes in EELV

**Fig. 3:**
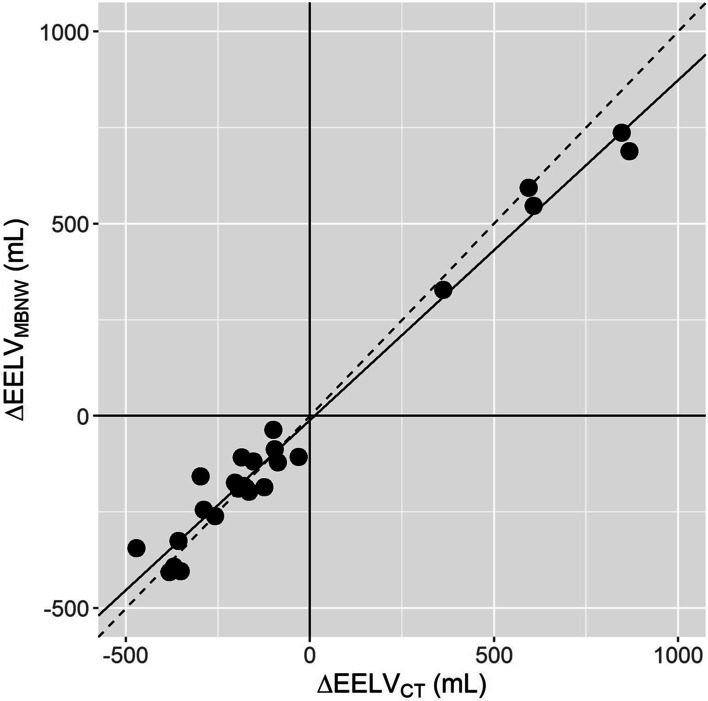
Four quadrants plot relating EELV_MBNW_ and EELV_CT_ changes between consecutive measurements. Horizontal and vertical continuous black lines are quadrant limits. Oblique dashed line is line of identity. Solid line is regression line. Each symbol is the change in end-expiratory lung volume between consecutive measurements assessed with the multiple breath nitrogen washin–washout technique (ΔEELV_MBNW_) and with computed tomography (ΔEELV_CT_)

**Fig. 4:**
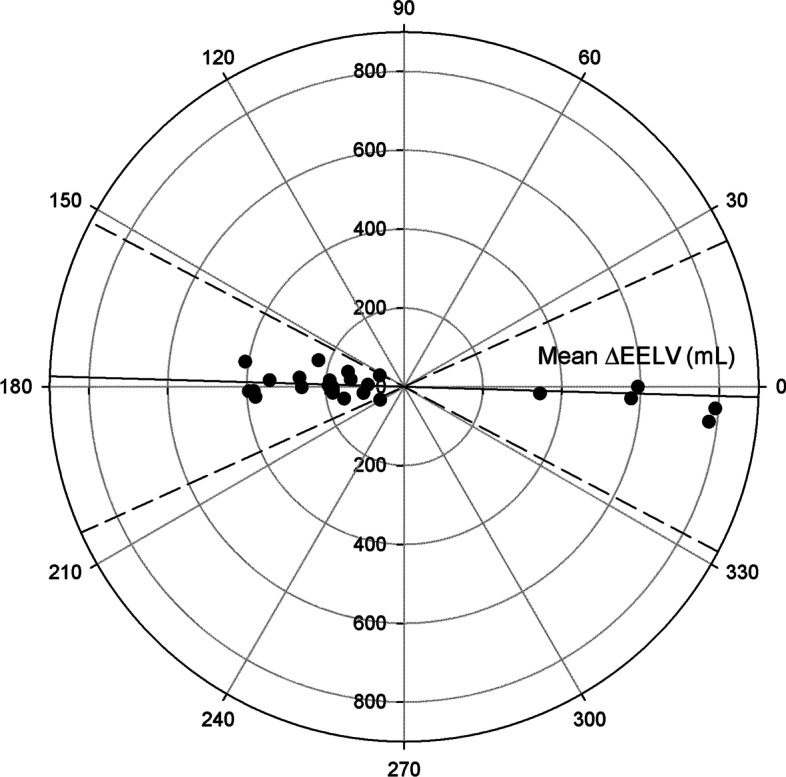
Polar plot assessing trending ability of EELV_MBNW_ to track changes in EELV_CT_. The radial axis joining 0 to 180° is a 45° clockwise rotation of the line of identity in the four-quadrant plot and represents perfect agreement. The better the agreement between ΔEELV measurements, the closer data pairs will lie along the horizontal radial axis. The distance from the center of each plot represents the mean change in EELV between methods (mean ΔEELV) at each consecutive time points. Data points located between 315 and 45° refer to time points in which both EELV_CT_ and EELV_MBNW_ increased (upper right quadrant of the four-quadrant plot), while data points located between 135 and 225° refer to consecutive time points in which both EELV_CT_ and EELV_MBNW_ decreased (lower left quadrant of the four-quadrant plot). Data points located between 45 and 135° or 225 and 315° correspond to disagreement in the directional change of EELV between both techniques. Continuous line represents angular bias, while dashed lines represent radial limits of agreement. EELV_MBNW_ = end-expiratory lung volume assessed with the multiple breath nitrogen washin–washout technique; EELV_CT_ = end-expiratory lung volume assessed by computed tomography; ΔEELV = change in EELV between consecutive measurements

EELV_MBNW_ adequately tracked EELV_CT_ changes over time (Fig. 3), with a concordance rate amounting to 100% [CI_95%_: 87%–100%]. Results of the polar plot analysis are reported in Fig. 4. The angular bias amounted to – 2° ± 10° and was not statistically different from 0 (*p* = 0.21). Radial limits of agreement amounted to ± 26°.

### Comparison of recruited volume assessed by the MBNW technique and CT

**Fig. 5:**
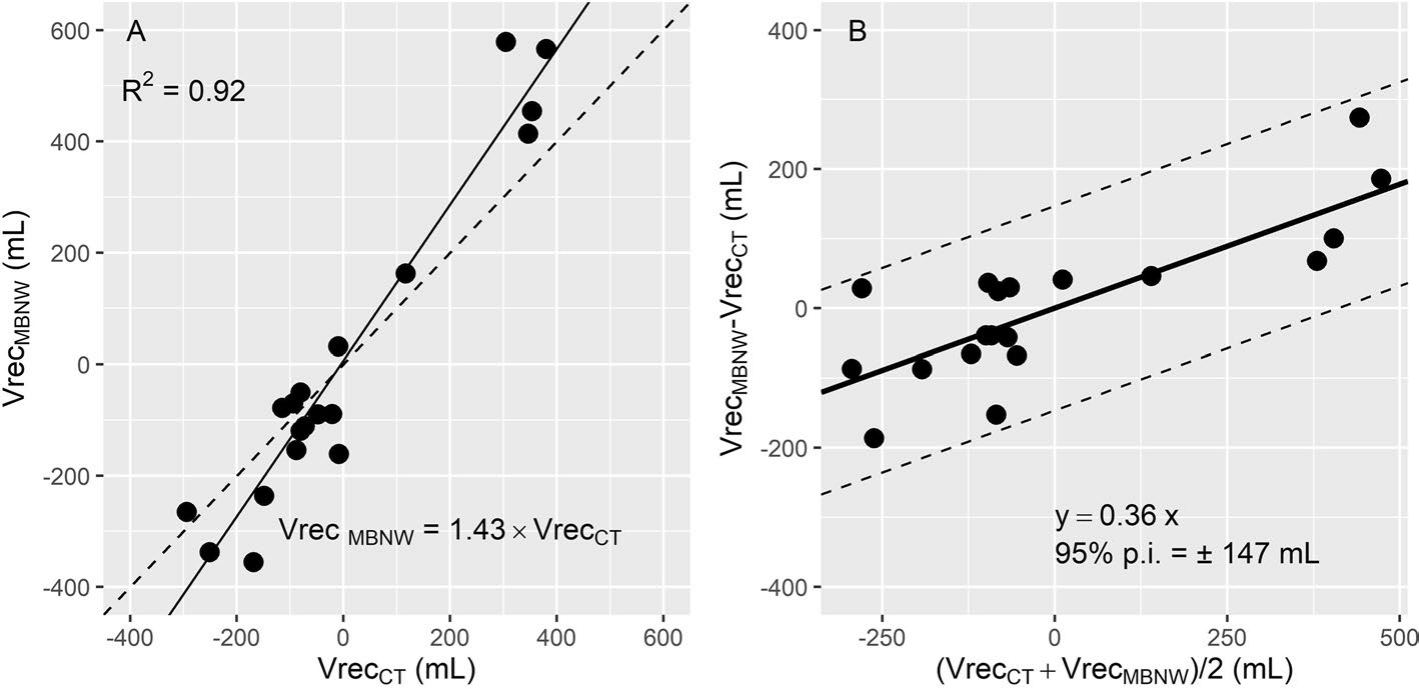
Correlation between Vrec_MBNW_ and Vrec_CT_ (**A**) and corresponding Bland and Altman plot (**B**). Each symbol represents a concomitant measurement of alveolar recruitment assessed with the multi-breath nitrogen washin–washout technique (Vrec_MBNW_) and computed tomography (Vrec_CT_). Negative recruited volumes denote alveolar derecruitment. **A**: Solid line is regression line. Dashed line is line of identity. **B**: continuous line and the 2 broken lines are the mean bias and 95% prediction interval limits of the bias between methods_,_ respectively. 95% p.i. = 95% prediction interval of the bias between methods

Vrec_MBNW_ and Vrec_CT_ were significantly correlated (marginal *R*^2^ = 0.92, *p* < 0.001, Fig. 5A), and the regression equation between EELV_MBNW_ and EELV_CT_, had an intercept of 0 mL (*p* < 0.001) and a slope of 1.43. A non-constant bias between methods was identified on Bland and Altman plot, slightly increasing toward more positive values as Vrec increased (Fig. 5B). The 95% prediction interval of the bias between methods amounted to ± 147 mL.

## Discussion

The main findings of the study are the following: (1) the new MBNW monitoring tool is reliable to assess EELV at bedside, with statistically significant trending ability; (2) the precision of the technique is good, with a percentage error against CT amounting to 13.7%, and a LSC between repeated measurements ≤ 10%; (3) estimation of alveolar recruitment from EELV_MBNW_ and measured with CT are highly correlated.

### ***Reliability and trending ability of EELV***_***MBNW***_

As previously reported, EELV assessed by the MBNW technique exhibited a systematic bias, as compared to EELV_CT_ [6], with a slight underestimation at high values of EELV. Although considered as a “gold standard” for lung volume assessment, it is important to highlight that CT scans accurately measures the “anatomical lung volume”, in contrast with MBNW technique, or other gas-dilution technique, where the lung volume measured contributes to ventilation, i.e., “ventilable lung volume” [4]. Thus, in the presence of overinflated lung areas (at high PEEP levels) the “anatomical lung volume” is expected to be different from the “ventilable lung volume” [7, 28]. The current study supports previous results, since the bias observed between the tested methods was independently related to the amount of overinflation at end-expiration, it may be hypothesized that overinflated lung areas did not participate to nitrogen mixing during washin–washout, which may explain EELV underestimation at high PEEP. From the clinical standpoint, the bias observed between techniques in the current study is likely to be of minor relevance if we consider the potential clinical application of the proposed novel technology and the drawbacks of the CT technique.

Nevertheless, the upper limit of the CI_95%_ of the LSC between repeated measurements amounted to 10%, suggesting a good ability to detect relatively small changes in EELV. This was confirmed by a 100% concordance rate and an angular bias not significantly different from 0, suggesting a good trending ability of the new technique. The percentage error of this new technique was the lowest of previously published experimental and clinical studies (ranging from 28 to 57% [4–6]). This is presumably related to the use of the fast mainstream sensors, which ensured that the measurements of proximal gas concentrations and gas flow are precisely aligned in time [29]. Signal alignment may be more challenging when a side stream sensor is used, as in a previous study using the same ARDS animal model [6]. One might argue that despite considerable improvement on EELV_MBNW_ accuracy provided by this novel technology, lower PEEP levels were used in the current study. However, the experimental protocol presented here is closer to the clinical setting in terms of applied PEEP levels, improving its extrinsic validity.

## Reliability of estimated alveolar recruitment by the MBNW technique

To our knowledge, the present study is the first to provide concomitant measurements of Vrec_MBNW_ with CT as the reference technique. As proposed by Dellamonica et al. [3], Vrec_MBNW_ is computed from the difference between change in EELV induced by PEEP, and minimally predicted increase in lung volume between PEEP levels (i.e., the product of respiratory system compliance assessed at lower PEEP and PEEP level change). When the change in EELV is larger than this minimum predicted volume gain, the difference is considered as an estimate of alveolar recruitment. However, this method assumes that compliance is linear over the change in pressure induced by PEEP; an assumption we are unable to confirm with our data. Furthermore, this method combines measurement errors from several devices (ventilator pneumotachograph, ventilator pressure sensors, MBNW technique), questioning the ability of this technique to detect small recruited volumes. Nevertheless, a fair correlation was identified between Vrec_MBNW_ and alveolar recruitment computed from the pressure–volume curves in ARDS patients [3], but mathematical coupling between both techniques (Vrec_MBNW_ and the pressure volume curves are computed using the ventilator pneumotachograph) could question the validity of this study. Of note, 4 subjects with the highest PEEP levels (16 cm H_2_O) were excluded of Dellamonica et al. study as their EELV_MBNW_ was underestimated [3], a finding in line with our results. Nevertheless, we observed in the present study a strong correlation with CT measurements, although Vrec_MBNW_ was overestimated at high recruited volume values (a consequence of the underestimation of EELV_MBNW_). Whether this technique could reliably detect low and high recruiters by PEEP remains to be determined.

### Clinical perspective

As compared to other MBNW technologies [4–7], the current monitoring tool exhibited substantially lower percentage error (28% to 57% vs 13.7%) and hence higher precision, and narrower radial limits of agreements (± 51° [6] vs. ± 26°), i.e., higher trending ability. Furthermore, the current monitoring tool is virtually unbiased, provided the conversion equation presented above is used to account for the non-constant bias [30]. Although we did not perform a direct comparison, we hypothesize that an improved accuracy could result from fast mainstream gas concentration measurements able to better accommodate the heterogeneities in the distribution of the ventilation than a device based only on end-tidal gas composition. The availability of a technology providing accurate bedside assessment and trending ability of end-expiratory lung volume allows foreseeing the regular assessment of a recruitment maneuver or prone positioning. Likewise direct assessment of the effects on EELV of a PEEP change should make PEEP optimization part of systematic daily practice. Furthermore, measurement of EELV allows computation of lung strain (the lung deformation related to its original status) and lung stress (using a presumed value for specific elastance), both being important parameters to evaluate the risk of ventilator-induced lung injury. This should help implement a personalized lung protective mechanical ventilation.

### Study limits

Some limitations of the present study should, however, be acknowledged. First, the experimental ARDS model used is highly recruitable by PEEP, and external validity in poorly recruitable ARDS may be questionable. Second, the reliability of the technique was not tested at VT lower than 6 ml.kg^−1^, at FiO_2_ higher than 80% and higher respiratory rates (*i.e*., situations in which the MBNW technique may be less reliable [6]). Furthermore, the present study was performed in deeply sedated animals under neuromuscular blocking agents and controlled ventilation, and the reliability of the present MBNW technique during mechanical ventilation with assisted ventilation modes remains unknown. Third, lung segmentation on CT excluded the trachea and the main bronchus (*i.e.,* structures involved in EELV computed by the MNBW technique), which could explain a slight underestimation of EELV_CT_, although this would be a systematic error as these structures are cartilaginous and are not expected to increase their volumes at increasing PEEP levels.

## Conclusions

We report a novel technique that reliably assesses EELV at bedside, and might add valuable information to further personalization of mechanical ventilation. This novel system designed to assess EELV shows high measurement precision and with excellent trending ability, compared to EELV estimated using CT, as the reference technique. Finally, estimation of PEEP-induced alveolar recruitment using this novel system appears reliable in the specific setting of a highly recruitable experimental ARDS model.

## Supplementary Information

**Additional file 1.** Available and missing measurements.

## Data Availability

The datasets used and/or analyzed during the current study are available from the corresponding author on reasonable request.

## Abbreviations

ARDS: Acute respiratory distress syndrome
CI_95%_: 95% Confidence interval
CT: Computed tomography
CV: Coefficient of variation
EELV: End-expiratory lung volume
FiO_2_: Inspired fraction of oxygen
LSC: Least significant change
MBNW: Multiple-breaths nitrogen washin–washout
PEEP: Positive end-expiratory pressure
RR: Respiratory rate
VCO_2_: Carbon dioxide production
VO_2_: Oxygen consumption
Vrec_CT_: Recruited alveolar volume assessed with CT
Vrec_MBNW_: Recruited alveolar volume assessed with the MBNW technique
VT: Tidal volume
