# Supplementary material for: Validation of a novel system to assess end-expiratory lung volume and alveolar recruitment in an ARDS model
Source: Intensive Care Med Exp. 2021 Sep 10;9:46. doi: 10.1186/s40635-021-00410-x (PMC8428961; doi:10.1186/s40635-021-00410-x)
Supplement: Supplementary file 1 — Additional file 1. Available and missing measurements. [file 40635_2021_410_MOESM1_ESM.docx]

**Additional file 1. Available and missing measurements**

|  | Baseline | Post-injury | PEEP#1 | PEEP#2 | PEEP#3 | PEEP#4 |
| --- | --- | --- | --- | --- | --- | --- |
| Pig#1 |  |  |  |  |  |  |
| Pig#2 |  |  |  |  |  |  |
| Pig#3 |  |  |  |  |  |  |
| Pig#4 |  |  |  |  |  |  |
| Pig#5 |  |  |  |  |  |  |
| Pig#6 |  |  |  |  |  |  |
| Pig#7 |  |  |  |  |  |  |

Green : available measurement; grey: missing measurement related to MBNW captor dysfunction; yellow: missing measurement related to excessive hypoxemia at the lowest PEEP level; red: missing measurements related to premature animal death.

PEEP=positive end-expiratory pressure; MBNW = multiple breath nitrogen washin-washout.

*
